# The Impact of the Early COVID-19 Global Pandemic on Children Undergoing Active Cancer Treatment and Their Parents

**DOI:** 10.3390/curroncol30020186

**Published:** 2023-02-17

**Authors:** Andrew Tran, Sharon H. J. Hou, Caitlin Forbes, Sara Cho, Victoria J. Forster, Mehak Stokoe, Claire E. Wakefield, Lori Wiener, Lauren C. Heathcote, Gisela Michel, Pandora Patterson, Kathleen Reynolds, Fiona S. M. Schulte

**Affiliations:** 1 Department of Oncology, Division of Psychosocial Oncology, Cumming School of Medicine, University of Calgary, Calgary, AB T2N 1N4, Canada; 2 Department of Psychology, British Columbia Children’s Hospital, Vancouver, BC V6H 3N1, Canada; 3 Arthur and Sonia Labatt Brain Tumour Research Centre, The Hospital for Sick Children, Toronto, ON M5G 1X8, Canada; 4 School of Women’s and Children’s Health, UNSW Medicine and Health, UNSW Sydney, Sydney, NSW 2052, Australia; 5 Kids Cancer Centre, Sydney Children’s Hospital, Randwick, NSW 2031, Australia; 6 Center for Cancer Research, National Cancer Institute, National Institutes of Health, Bethesda, MD 20892, USA; 7 Health Psychology Section, Department of Psychology, Institute of Psychiatry, Psychology and Neuroscience, King’s College London, London WC2R 2LS, UK; 8 Department of Health Sciences and Medicine, University of Lucerne, 6002 Lucerne, Switzerland; 9 Research, Evaluation and Policy Unit, Canteen Australia, Sydney, NSW 2042, Australia; 10 Faculty of Medicine and Health, University of Sydney, Sydney, NSW 2006, Australia; 11 Long Term Survivor’s Clinic, Alberta Children’s Hospital, Calgary, AB T3B 6A8, Canada; 12 Department of Medicine, Faculty of Family Medicine, University of Calgary, Calgary, AB T2N 1N4, Canada

**Keywords:** pediatric oncology, psychosocial oncology, mental health, COVID-19, active treatment

## Abstract

(1) Background: The COVID-19 global pandemic has impacted people worldwide with unique implications for vulnerable groups. In this cross-sectional study, we examined the impact of the early pandemic on children undergoing active cancer treatment and their parents. (2) Methods: In May 2020, 30 parents of children undergoing active cancer treatment completed an online survey regarding the impact of COVID-19 on their child’s cancer care, perceived utility of telemedicine, and child and parent mental health status. (3) Results: Most participants (87%) reported that they did not experience any changes to major cancer treatments. Among those who reported using telemedicine, 78% reported this to be *beneficial*. Over half of the participants reported that their child’s mental health status was worse now than prior to the COVID-19 global pandemic. Parent-reported child anxiety scores were significantly higher for those who reported changes to mental health care for their child compared to those who did not report the same, *t*(25.99) = −3.04, *p* = 0.005. (4) Conclusion: Child and parent mental health status were affected when compared to pre-pandemic. Telemedicine appears to be a promising complement to face-to-face meetings for some families and warrants further exploration.

## 1. Introduction

Each year, globally, approximately 300,000 children aged 0–14 years are diagnosed with cancer [1]. The means needed to successfully treat cancer, however, can produce toxicity and lead to additional health complications (e.g., disability) for children with cancer, impairing their quality of life [2,3]. Fortunately, survival rates of childhood cancers have increased substantially in recent years due to refinements in cancer treatments. As of 2021, 84% of children living in high-income countries survive at least five years post-diagnosis, which is an increase from approximately 70% in the 1980s [4,5]. Examples of chronic health problems stemming from cancer treatments include, but are not limited to, fatigue, nausea, pain, social isolation, and immunosuppression [6,7,8]. Additional health concerns may also include social and psychological challenges (e.g., anxiety and depression) [9,10,11,12,13,14].

The COVID-19 global pandemic is caused by the severe acute respiratory syndrome coronavirus 2 (SARS-CoV-2) [15]. As of June 2022, COVID-19 has infected over 535 million people worldwide, resulting in significant morbidity and mortality [16]. Guidelines published by pediatric oncology organizations such as the Children’s Oncology Group (COG) have urged children with cancer and their families to take extra precautions amid the early COVID-19 global pandemic [17]. Recent research suggests that despite being immunocompromised, children with cancer are not at increased risk for a severe course of COVID-19 for those living in high-income countries [18,19,20]. In addition to the perceived risks of infection for COVID-19, there have been nonetheless considerable changes and impacts to the care and mental health of children with cancer and their families due to the COVID-19 global pandemic [21,22]. 

Children on active cancer treatment and their families oftentimes experience enduring treatment protocols with treatment-related difficulties (e.g., side effects) [23,24]. As such, this vulnerable group faces unique burdens related to their care in the context of the COVID-19 global pandemic. Studies conducted early on in the COVID-19 global pandemic suggest that there is a need to better balance navigating the risk of the COVID-19 global pandemic with ensuring continuity of cancer care and healthy living (e.g., adequate physical activity and social interaction) for children with cancer [25,26]. More recently, research on the impact of the COVID-19 global pandemic on children with cancer showed that the majority of parents (85%) of children with cancer reported worries surrounding the COVID-19 global pandemic [27]. Furthermore, children on active treatment in particular often endure treatment protocols spanning year-long periods. In the early days of the COVID-19 global pandemic, these children and their families faced sudden and drastic changes to the format in which they received treatments and attended appointments, including the use of telemedicine [28,29,30,31]. Therefore, understanding the utility of telemedicine for children on active cancer treatment and their families at the beginning of the COVID-19 global pandemic may offer insights into how healthcare providers were offering support to this vulnerable group in their care at the peak of uncertainty.

Research suggests that the use of telemedicine in the context of delivering health care in the COVID-19 global pandemic appears promising. Specifically, some studies showed that the uptake of telemedicine has been well received by general healthcare providers and parents of immunocompromised children for several reasons, including increased accessibility, lowered financial burden, and lowered risk of COVID-19 infection [32,33]. Other studies indicate that, throughout the COVID-19 global pandemic, healthcare providers and patients, including adult survivors of cancer and general pediatric patients, have reported high levels of satisfaction with the use of telemedicine with some healthcare providers appreciating the use of telemedicine in care moving forward [34,35,36]. In fact, some research suggests that virtual care (i.e., medical care, physical care, and health behavior interventions using telemedicine) may be delivered sustainably for survivors of childhood cancer during the pandemic and moving forward [37]. Importantly, a few studies have investigated the use of telemedicine for children and families on active treatment for their health. Specifically, one study found that telemedicine provides similar or improved health outcomes compared to face-to-face meetings for children with hematologic conditions undergoing treatment [38]. Two other studies on pediatric telemedicine including children with cancer more broadly have found that telemedicine has been associated with good diagnosis accuracy, improving care management, lowering mortality, and reducing inaccessibility to specialists and resources [39,40].

Beyond their cancer care, there is also a need to better understand how children on active cancer treatment and their parents are managing from a mental health perspective. Existing literature has mostly focused on the unique medical and mental health needs of children in palliative care or survivorship, or adolescent and young adult patients [22,41,42,43,44,45,46,47]. For example, during the COVID-19 global pandemic, adolescent and young adult (AYA) survivors of childhood cancer reported worsened mental health status that was linked to their past cancer experience [43]. In another study, Kosir and colleagues (2020) found that AYA patients and survivors reported increased psychological distress compared to pre-pandemic [44]. Together, this literature suggests that there are adverse mental health impacts on young people in their survivorship. However, very little work has focused exclusively on exploring the mental health status of children on active cancer treatment and their parents. Notably, Alshahrani and colleagues (2020) conducted a cross-sectional survey in Saudi Arabia to examine the experiences of children undergoing active cancer treatment in a tertiary care institution. The authors found that children patients reported heightened feelings of anxiety, anger, depression, and fear during the COVID-19 global pandemic [48].

Over two and a half years have passed since the onset of the COVID-19 global pandemic. The ongoing and evolving nature of the COVID-19 global pandemic continues to impact people worldwide and particularly those from vulnerable groups. There is a need to fully capture the impact of the early COVID-19 global pandemic on children on active cancer treatment and their parents to better understand the changes that have occurred in their care experience since then and determine supports that can be used to address their needs today and moving forward. This study was unique in that we sought to understand the experiences of both parents and their child undergoing active cancer treatment from the perspective of the parents. Specific objectives for this study are stated below.

Objective 1. To explore changes (i.e., delayed, modified, canceled) in health care for children on active treatment, including their major cancer care (e.g., chemotherapy, surgery) and mental health care. 

Objective 2. To explore the perceived utility (benefits and effectiveness) of telemedicine.

Objective 3. To describe the mental health status of children on active cancer treatment and their parents. 

Objective 4. To examine whether cancer treatment changes and perceived utility of telemedicine (i.e., perceived effectiveness when compared to face-to-face) are associated with child and parent mental health status.

## 2. Materials and Methods

### 2.1. Recruitment

Recruitment was conducted through a multipronged approach. We used social media (Twitter and Facebook) to engage with children on active cancer treatment across Canada and the United States. Patient advocacy groups and partner organizations, such as Kids Cancer Care, also facilitated recruitment through convenience and snowball sampling. 

### 2.2. Participants

This study was conducted as part of a larger study on COVID-19 and childhood cancer. Inclusion criteria for the parent participants required that their children were: (1) under the age of 18 years at the time of study; and (2) undergoing active cancer treatment. There were no other restrictions to increase the representativeness of the sample and generalizability.

### 2.3. Procedure

The current study adhered to the Strengthening the Reporting of Observational Studies in Epidemiology (STROBE) [49]. Please refer to Supplementary Materials for the STROBE statement. Between May and June 2020, we conducted a quantitative cross-sectional study with parents of children undergoing active cancer treatment. We stopped recruiting after two months to solely capture the early phase of the COVID-19 global pandemic. Given the exploratory nature of this study, we did not conduct a power analysis to determine a sample size goal. The experiences of children with cancer and their parents were gathered through parent reports. Parent participants were asked to complete a battery of online questionnaires examining demographic information, the impact of the COVID-19 global pandemic, and psychosocial outcomes. The questionnaires and informed consent were administered using REDCap, a data management tool hosted at the University of Calgary [50,51]. This study was approved by the Health Research Ethics Board of Alberta—Cancer Committee (HREBA.CC-20-0151). 

### 2.4. Measures

#### 2.4.1. Demographic and Clinical Information

We collected demographic information on the child, which included age, sex, gender, country of residence, and living arrangement. Demographic information on the parent and family unit was also collected, including relationship to the child, gender, current relationship status, level of education, ethnicity, and household income. Clinical information on the child, including diagnosis, date of diagnosis, relapse, and primary country of treatment was gathered.

#### 2.4.2. COVID-19 and Cancer Questionnaire

We developed a 26-item questionnaire to assess the impact of the COVID-19 global pandemic on participants based on the multidisciplinary expertise of the research team in medicine, pediatric oncology, psychology, and psychosocial oncology; see Table S1. Participants were asked to respond to questions in a combination of multiple choice, yes/no, checkbox, and open-text format. Items assessed the perceived impact of the COVID-19 global pandemic on medical treatments (e.g., “Have any type(s) of your child’s cancer treatments been affected as a result of COVID-19?”) and the effectiveness of telemedicine (e.g., “Did you feel the telemedicine meeting was as effective as a face-to-face meeting?”) on parents and their child’s treatment experience. Parents self-reported on whether they believed their child’s treatments were affected by the COVID-19 global pandemic: “Not affected”, “Delayed”, “Modified”, “Cancelled”, or “Not applicable”. The questionnaire also examined factors that parents believed impacted their child’s current mental health status (e.g., “Is your child’s current mental health tied to… their past fears/worries about their past cancer treatment?”). See Supplementary Materials (Table S1) for the complete questionnaire.

#### 2.4.3. Mental Health Status

Anxiety and depression were measured using the Patient Reported Outcomes Measurement Information System (PROMIS Profile-29) anxiety and depression subscales [52]. Parents completed a self-report and then a parent-proxy for their child. The anxiety and depression measures were four-item scales rated on a five-point Likert scale (1 = never, 5 = always). Raw scores were summed and converted to a standardized t-score with a mean of 50 and standard deviation of 10 based on an American population sample. *T*-scores were interpreted according to the following: <55: none to slight; 55–59: mild; 60–69: moderate; 70+: severe [53]. The PROMIS has been validated for use in pediatric oncology for 8 to 17 years of age [54]. Internal consistency for this sample for parent-proxy anxiety was excellent (α = 0.93); internal consistency for parent-proxy depression was good (α = 0.89). Internal consistency for parent self-report anxiety (α = 0.90) and depression (α = 0.92) was excellent.

### 2.5. Statistical Analyses

Data analyses were performed using IBM SPSS 25 [55]. To address Objectives 1 and 2, we computed frequencies for changes to health care and perceived utility (benefits and effectiveness) of telemedicine. To address Objective 3, we computed frequencies and descriptives for responses on parent and child mental health status (i.e., anxiety and depression). To address Objective 4, we conducted independent samples *t*-tests to examine mental health status (i.e., anxiety and depression) when comparing parents’ perceived effectiveness of telemedicine. We also performed an independent samples *t*-test to compare the impact on health care (i.e., mental health treatment) and parent/child anxiety and depression *t*-scores. Pairwise deletion was used to handle missingness in analyses [56].

## 3. Results

### 3.1. Descriptive and Clinical Characteristics

A total of 30 parents of children on active cancer treatment completed the study. Parent participants predominantly identified as the biological mother (*n* = 25, 83%). In addition, 63% (*n* = 19) of parents and their child lived in Canada, while the remainder lived in the United States (*n* = 11, 37%). The mean age of children undergoing active cancer treatment was 8.79 years (*SD* = 5.10). Furthermore, their mean age at diagnosis was 7.23 years (*SD* = 5.05), and their mean time since diagnosis was 2.34 years (*SD* = 2.27). Sex of the children was 47% male (*n* = 14) and 53% female (*n* = 16). Acute Lymphoblastic Leukemia was the most common cancer diagnosis (*n* = 17, 57%). Descriptive characteristics of the sample can be found in Table 1.

### 3.2. Objective 1: Changes (Delays, Modifications, Cancellations) to Health Care

**Figure 1:**
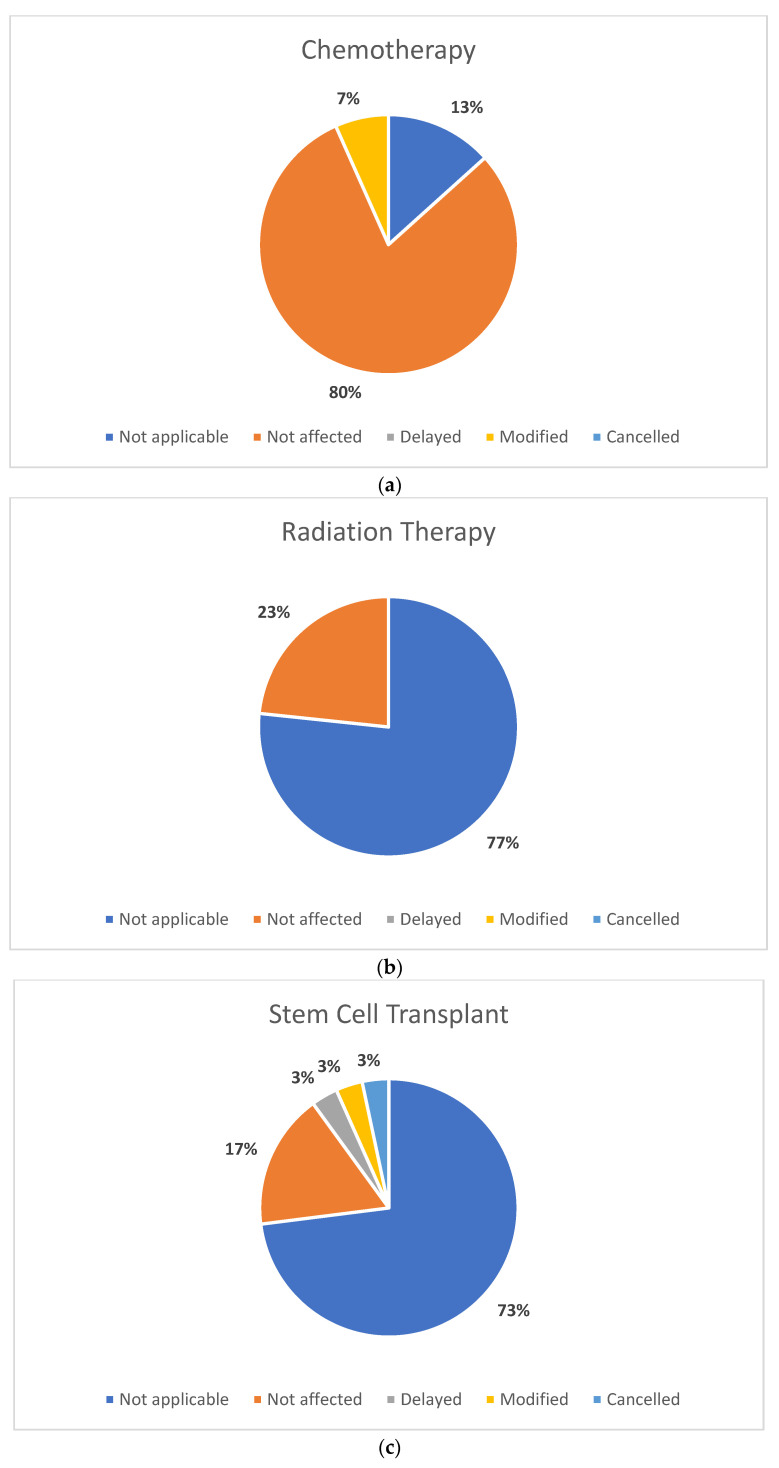

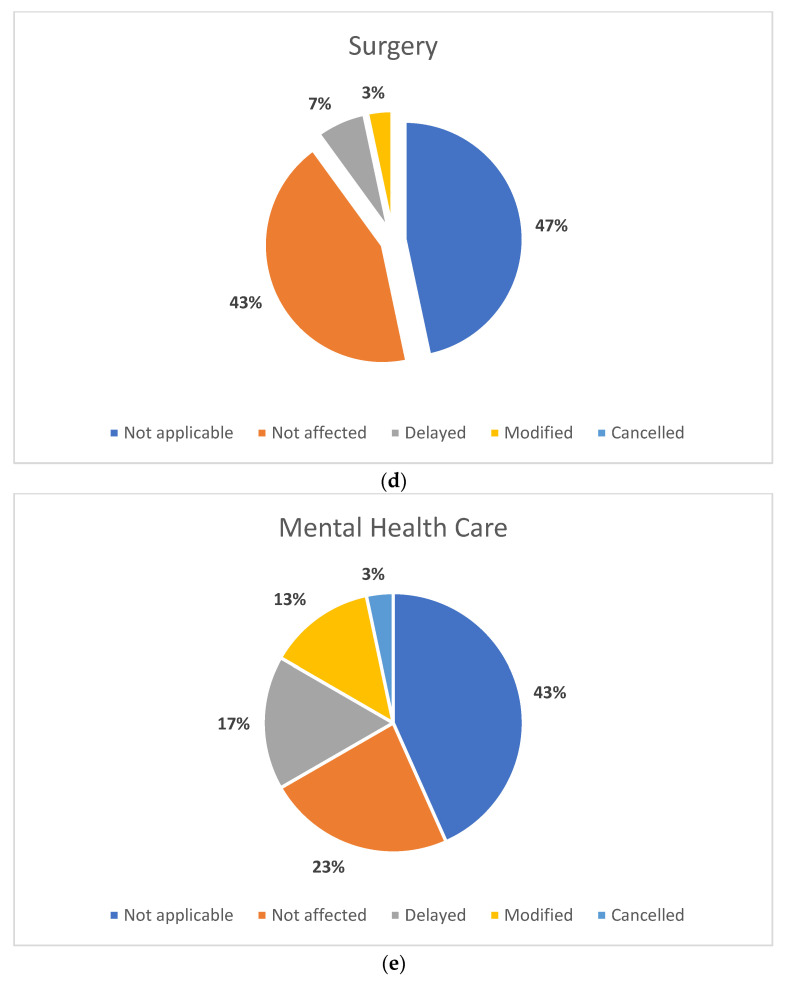
Parents self-reported disruptions to their child’s health care treatments according to their own interpretation of “Delayed”, “Modified”, and “Cancelled”: (**a**) chemotherapy; (**b**) radiation therapy; (**c**) stem cell transplant; (**d**) surgery; (**e**) mental health care.

Participants were asked to report on changes they experienced with different types of care, including cancer care, mental health care, and routine investigations, for their child. Most participants (*n* = 26, 87%) indicated that their child did not experience any changes to major cancer treatments, such as chemotherapy, radiation therapy, stem cell transplant, and surgery. Some participants (*n =* 13, 4%) reported changes to cancer care. Specifically, 10% (*n* = 3) of participants reported changes to their stem cell transplant (*n* = 1; 3% delayed, *n* = 1; 3% modified, *n* = 1; 3% canceled), 10% (*n* = 3) of participants reported changes to their surgery (*n =* 2; 7% delayed, *n =* 1; 3% modified), and 7% of participants reported changes to their (*n* = 2) chemotherapy (i.e., modified); see Figure 1 for a full summary. Furthermore, 59% (*n* = 10) of participants reported a change in their child’s access to mental health care due to the COVID-19 global pandemic. Specifically, of those children currently receiving mental health treatment (total *n* = 17), 29% (*n* = 5) were *delayed*, 24% (*n* = 4) were *modified*, and 6% (*n* = 1) were *canceled*. Despite report of this disruption, 79% (*n* =23) of participants nonetheless indicated that they believed that their child received the necessary psychosocial support. Participants were also asked to report on whether they believed that, as parents/guardians, they received adequate psychosocial support; 69% (*n* = 20) indicated that they received the support they needed in this regard. We are missing data from one participant for the two items reporting on whether the child/parent received adequate psychosocial support.

### 3.3. Objective 2: Perceived Utility (Benefit and Effectiveness) of Telemedicine

Participants were asked to report on their perception of the benefit of telemedicine. Of the total sample, 31% (*n* = 9) of participants had accessed telemedicine to complement traditional face-to-face meetings at their hospital clinics. Among these respondents, most (89%; *n* = 8) perceived telemedicine to have *little benefit* to being *very beneficial*. One participant did not report on one item related to perceived benefit of telemedicine.

Participants were also asked to report on the perceived effectiveness of telemedicine compared to face-to-face meetings. Most respondents (78%; *n* = 7) viewed telemedicine as *somewhat effective* or just *as effective as face-to-face* meetings. No respondents viewed telemedicine as *very effective*, *better than face-to-face* meetings (see Table 2).

### 3.4. Objective 3: Child and Parent Mental Health Status

Parents reported on their child’s mental health status. Twenty-one percent (*n* = 6) of parents reported that their child’s mental health status was *much worse* now compared to pre-pandemic and 35% (*n* = 10) believed it was *somewhat* worse now. Moreover, nearly half of the parents (*n* = 13, 45%) believed that their child’s mental health status was *about the same* now as before the pandemic. No parents reported that their child’s mental health status was *somewhat better* or *much better* now. The most common reasons contributing to child mental health status included feelings of isolation from peers (*n* = 24, 80%), fears/worries about their cancer and treatment (*n* = 15, 50%), difficulties with remote learning (*n* = 12, 40%), fears/worries about catching COVID-19 (*n* = 9, 30%), and fears/worries about uncertainty related to the COVID-19 global pandemic (*n* = 9, 30%; see Table 3).

Parents also reported on their own mental health status with approximately one-quarter of the sample (*n* = 7, 24%) of participants indicating their mental health status is *much worse* now as compared to pre-pandemic, and a greater proportion of participants (*n* = 18, 62%) reported that it was *somewhat worse* now. Fourteen percent (*n* = 14) of parents reported their mental health status to be *about the same*. No parents reported that their mental health status was *somewhat better* or *much better* now compared to pre-pandemic. The most prevalent reasons contributing to parent mental health status included fears/worries about their child’s cancer and treatment (*n* = 26, 87%), fears/worries about the uncertainty related to how COVID-19 might impact their child with cancer (*n* = 25, 83%), fears/worries about catching COVID-19 (*n* = 22, 73%), and fears/worries about uncertainty related to the COVID-19 global pandemic (*n* = 22, 73%; see Table 3). One participant did not report on two items related to mental health status.

### 3.5. Objective 4: Impact of Health Care Changes and Perceived Utility of Telemedicine on Child and Parent Mental Health Status

We examined the mental health status (i.e., anxiety and depression) of children and their parents in relation to changes reported in the child’s health care (i.e., mental health care: change, no change) in the context of the COVID-19 global pandemic. Independent samples *t*-test revealed that parent-reported child anxiety scores were significantly higher for participants that indicated changes in mental health care for their child (*M* = 61.33, *SD* = 6.80) compared to those who reported no changes in mental health care for their child (*M* = 50.40, *SD* = 12.26), *t*(25.99) = –3.04, *p* = 0.005, *d* = 1.02. Results are displayed in Table 4.

We also examined the mental health status (i.e., anxiety and depression) of children and their parents in relation to the perceived utility of telemedicine (i.e., perceived effectiveness of telemedicine compared to face-to-face meetings: as effective as face-to-face, not as effective as face-to-face). Participants who accessed telemedicine and perceived it to be as effective as face-to-face meetings reported significantly lower child depression scores than those who did not report this to be the case, *t*(7) = 3.34, *p* = 0.012, *d* = 2.68. Participants who accessed telemedicine and perceived it to be as effective as face-to-face meetings reported significantly lower parent anxiety scores, *t*(7) = 3.43, *p* = 0.011, *d* = 2.75, and lower parent depression scores, *t*(6.55) = 5.07, *p* = 0.002, *d* = 2.35 than those who did not perceive this to be the case. Results are found in Table 5. Two participants did not report on both parent and child anxiety and depression measures. One participant did not report on parent anxiety and depression measures.

## 4. Discussion

The COVID-19 global pandemic has affected, and continues to affect, children and families worldwide. The current work offers an important insight into the experiences of children on active cancer treatment and their parents in the context of an unprecedented time with peak levels of uncertainty, including changes in health care, mental health status, and use of telemedicine.

Parents reported minimal disruptions to major cancer care needs, including surgery, chemotherapy, and radiation. This is likely reflective of efforts made by treatment centers to prioritize major treatments for children. However, parents reported changes to mental health care for their children on active cancer treatment. These findings contribute to existing work that have documented the varying degrees of disruptions observed in cancer care (e.g., reduced services leading to delays and disruptions of cancer care) in the ongoing COVID-19 global pandemic [31,57,58]. Importantly, the ramifications of changes to mental health care have yet to be fully understood in the context of the ongoing COVID-19 global pandemic. Our study showed that despite significant interruptions to mental health care, most parents believed that they and their child continued to receive adequate psychosocial support.

Parents found telemedicine to be generally beneficial and comparable in effectiveness when compared to face-to-face care. This finding is consistent with existing research showing that the uptake of telemedicine has been well received and used quite prevalently in the ongoing COVID-19 global pandemic [59]. Indeed, consistent with our results, there are many potential benefits to telemedicine, including increased access [32,60] and reach, for example, to rural or remote settings. There is therefore value in continuing to explore the use of telemedicine in health care.

Interestingly, no participants endorsed that telemedicine was better than face-to-face meetings. It can be suggested that providing care through telemedicine alone may not be sufficient. Rather, our data suggest that at the minimum, telemedicine may be used as a complement to face-to-face care. Children with cancer are often immunocompromised and at higher risk of viral illnesses. The onset of the COVID-19 global pandemic certainly exacerbated these pre-existing risks and concerns. Perhaps, with the emergence of telemedicine, this type of service delivery may serve as a viable intervention that complements existing face-to-face care moving forward [45,46,61]. Alternatively, perceptions of telemedicine may have changed since the start of the pandemic. It is possible that patients and their families and healthcare providers alike needed some time to acclimatize to telemedicine as part of clinical care and that a different perspective has emerged since the early stages of the COVID-19 global pandemic [62]. Ultimately, notwithstanding the COVID-19 global pandemic, the use of this complementary intervention may be worthwhile exploring in improving capacity for and access to care worldwide.

On the whole, parents reported poorer child and parent mental health status in the early phases of the COVID-19 global pandemic than prior to the onset of the COVID-19 global pandemic. This is consistent with research on the mental health of AYA with cancer [44]. Notably, many parents reported that their own mental health status was affected more than they reported that their child’s mental health was affected. The reasons identified by parents regarding their worsened mental health status were primarily tied to the COVID-19 global pandemic and uncertainty surrounding it as well as the subsequent social isolation. These findings are the first to examine the mental health status of parents along with children on active treatment, thereby highlighting the possible dyadic nature of the parent and child experience. Moreover, the overall reported increase in anxiety and depression in some parents and children is concerning and warrants an appropriate response because children on active cancer treatment have specific needs and limitations on their normal, everyday activities. It is also particularly concerning when parents also reported changes to their mental health care, which may have implications for their experience of poor mental health.

Children with cancer on active treatment and their parents face unique challenges in addition to the ongoing, daily difficulties experienced by all families worldwide in living through the COVID-19 global pandemic. For example, common stressors faced by all may include household financial difficulties and managing physical and mental health. Stressors specific to children with cancer on active cancer treatment and their parents may include balancing disruptions to health care and managing COVID-19 related risks. These are complex matters that likely add to the burden of care for parents of children on active treatment, therefore contributing to poorer mental health for parents than children. Furthermore, it is possible that the period of May to June 2020 when the study was conducted was particularly difficult for families as it was early in the COVID-19 global pandemic with widespread medical, psychological, social, and economic uncertainty. Understandably, experiences of social isolation may have been particularly anxiety provoking. These findings appear to align with other research conducted early in the global pandemic showing that the majority of parents (85%) of children with cancer were worried about COVID-19 [27].

Perceived effectiveness of telemedicine compared to face-to-face meetings (as effective as face-to-face, not as effective as face-to-face) was related to parent report of their child’s mental health status. This finding offers a unique contribution to the literature by highlighting the perceived effectiveness of telemedicine in the context of psychosocial and health care delivery during the COVID-19 global pandemic [63,64]. Previous studies have similarly found that a range of clinical services has been successfully delivered through telemedicine to adolescents and young adults with cancer. Our findings build on this by showing that telemedicine can be well received by parents of children on active cancer treatment.

Mental health status is linked to use of telemedicine and changes to mental health care. The mental health status of some children undergoing active cancer therapy and their parents may have deteriorated in the early stages of the COVID-19 global pandemic with disruptions to treatment or when traditional care is not possible. The perceived impacts we explored as reported by parents of children on active cancer treatment may continue to play a role today, two years later. It is possible that the mental health status of this group may be worse now due to the chronicity of the uncertainty and prolonged isolation. Alternatively, their mental health status may have improved as youth and families adjusted to living with the COVID-19 global pandemic. Telemedicine appears to be a promising tool in providing continuing care to these families; for example, by ensuring that families are receiving adequate resources and supports for mental health. Determining how telemedicine will be integrated with face-to-face care will be informative in future research as we return to more in-person visits. Perhaps by addressing any barriers impacting the delivery of telemedicine, healthcare providers may be able to better address mental health concerns for these children and parents by building greater accessibility to care for all families.

## 5. Limitations

There are several important limitations to the current study. The lack of a control/comparison group needs to be accounted for when interpreting the impacts of the COVID-19 global pandemic on the experiences of children undergoing active treatment and their parents. For instance, it is possible that the COVID-19 global pandemic contributed to poorer mental health status for all individuals, and thus, this finding may not be unique to children on active cancer treatment and their parents. Furthermore, the small sample size collected over the course of the recruitment period limits the generalizability of these findings to larger, diverse samples as the COVID-19 global pandemic continues to evolve. Future research collecting data from a larger sample is necessary to replicate the current work. Additionally, our recruitment strategy relied primarily on social media, which may reflect a biased sample selection. For example, most of our parent participants identified as White, mothers, coming from a high socioeconomic status, and spoke English. It can also be suggested that those most impacted by the COVID-19 global pandemic may be most motivated to participate in the studies. It is a prevailing issue in recruitment for psychosocial oncology research to lack representation from those from diverse social and cultural backgrounds [65]. Emphasizing recruitment through those from historically excluded groups may attenuate help to increase the representativeness of future studies. Finally, the current research employed a cross-sectional design, which limited our ability to capture the experiences of children on active cancer treatment and their families to one instance in time. Furthermore, the data collected reflected the early phases of the COVID-19 global pandemic. These early phases may have been particularly distressing for families because of the widespread uncertainty and fear of COVID-19. For these reasons, as research on the impacts of the COVID-19 global pandemic on health care continues to grow, it would be valuable to collect longitudinal data on the experiences of children on active cancer treatment and their families to capture change over time and in the context of the ongoing COVID-19 global pandemic.

## 6. Conclusions

The current research investigated how the early COVID-19 global pandemic has impacted children undergoing active cancer treatment and their parents from the parent’s perspective. While major cancer care was not delayed in this context, mental health care was clearly affected. Likewise, parent and child mental health status has worsened with the onset of the COVID-19 global pandemic. Telemedicine appears to be a promising format for the delivery of treatment that may complement face-to-face meetings. These results helped to capture the early experience of children on active cancer treatment and their parents, which will inform future research assessing how this vulnerable population is faring now over two years later.

## Figures and Tables

**Table 1:** Demographics Table of Children Undergoing Active Treatment with Parental Information.

Participant Demographics		*n* (%)	*M* (*SD*)
Participants			
	Total *N*	30	
Age (in years)			
	Current Age		8.79 (5.10)
	Age at Diagnosis		7.23 (5.05)
	Time since Diagnosis		2.34 (2.27)
Sex			
	Male	14 (46.7%)	
	Female	16 (53.3%)	
Ethnicity			
	White/European	28 (93.3%)	
	Black/African/Caribbean	2 (6.7%)	
	Latin American	2 (6.7%)	
	East Asian	1 (3.3%)	
	South Asian	1 (3.3%)	
	Other	1 (3.3%)	
Parent’s Education			
	Some high school or less	0 (0%)	
	High school	2 (6.7%)	
	Vocational school or training other than college/university	5 (16.7%)	
	Some university/college, no degree	5 (16.7%)	
	University/college, Bachelors degree	15 (50%)	
	Graduate/Professional degree	3 (10%)	
Household Income			
	Low income	2 (6.7%)	
	Low/middle income	6 (20%)	
	Middle income	13 (43.3%)	
	Middle/high income	4 (13.3%)	
	High income	1 (3.3%)	
	Prefer not to answer	4 (13.3%)	
Has your household’s income changed because of the COVID-19 pandemic?			
	No	14 (46.7)	
	Yes, my household income has decreased	16 (53.3)	
	Yes, my household income has increased	0 (0)	
Has your family had difficulty accessing food or other necessities due to the COVID-19 pandemic?			
	No	25 (83.3)	
	Yes	5 (16.7)	

Note. Basic demographic features of the children undergoing active cancer treatment and information about their parents and household.

**Table 2:** Parent Report of Telemedicine Use, Perceived Benefit, and Perceived Effectiveness.

Telemedicine Use		*n* (*%*)
Did you or your child participate in a telemedicine appointment(s)?		
	No	20 (69)
	Yes	9 (31)
Please rate your perception of the benefit of telemedicine		
	No benefit	1 (11.1)
	Little benefit	1 (11.1)
	Beneficial	5 (55.6)
	Very beneficial	2 (22.2)
Did you feel the telemedicine meeting was as effective as a face-to-face meeting?		
	Not at all	2 (22.2)
	Somewhat effective	5 (55.6)
	Effective	2 (22.2)
	Very effective, better than face-to-face	0 (0)

Note. Parent report of telemedicine use, perception of how beneficial it was (from no benefit to very beneficial), and if they felt that telemedicine was as effective as a face-to-face meeting (from not at all to very effective, better than face-to-face).

**Table 3:** Parent Report of Perceived Impact of Treatment Changes on Child and Parent Mental Health Status.

Child and Parent Mental Health Status	*n* (%)
Is your child’s current mental health impacted by…	
Feelings of isolation from peers	24 (80)
Fears/worries about their cancer and treatment	15 (50)
Difficulties with remote learning	12 (40)
Fears/worries about catching COVID-19	9 (30)
Fears/worries about the uncertainty related to the COVID-19 pandemic	9 (30)
Fears/worries about the uncertainty related to how COVID-19 might impact them as a survivor of childhood cancer	6 (20)
The media and messaging about the COVID-19 pandemic	5 (16.7)
Not applicable, my child’s mental health has not been impacted by the COVID-19 pandemic	3 (10)
Other	3 (10)
Is your current mental health impacted by…	
Fears/worries about your child’s cancer and treatment	26 (86.7)
Fears/worries about the uncertainty related to how COVID-19 might impact them as a child with cancer	25 (83.3)
Fears/worries about catching COVID-19	22 (73.3)
Fears/worries about the uncertainty related to the COVID-19 pandemic	22 (73.3)
Fears/worries about other factors in your life (i.e., boredom, feeling stuck at home)	21 (70)
Feelings of isolation from peers	19 (63.3)
Difficulties with remote learning	11 (36.7)
The media and messaging about the COVID-19 pandemic	8 (26.7)
Other	5 (16.7)
Not applicable, my mental health has not been impacted by the COVID-19 pandemic	0 (0)

Note. Parent report of factors that may have contributed to the perceptions of their child and their own current mental health status. Parents were allowed to select all that apply.

**Table 4:** Anxiety and Depression in Relation to Changes to Treatment.

	Delayed, Modified, Cancelled Treatment		No Change to Treatment						95% Confidence Interval	
	*n*	*M* (*SD*)	*n*	*M* (*SD*)	*t*-Test	df	*p*	Cohen’s *d*	LL	UL
Child Anxiety	10	61.33 (6.80)	18	50.40 (12.26)	−3.04	25.99	0.005	1.02	−18.33	−3.53
Child Depression	10	61.55 (6.21)	18	55.88 (12.83)	−1.57	25.72	0.128	0.52	−13.09	1.74
Parent Anxiety	9	58.56 (6.71)	18	61.26 (9.18)	0.78	25	0.441	0.32	−4.41	9.82
Parent Depression	9	53.21 (8.46)	18	55.51 (10.44)	0.57	25	0.572	0.23	−5.98	10.58

Note. Results from *t*-tests examining parent reports of child and parent mental health status (anxiety and depression) with impact on of health care changes (delayed, modified, cancelled vs. no change).

**Table 5:** Anxiety and Depression in Relation to Perceived Utility of Telemedicine.

	Telemedicine Is as Effective as Face-to-Face		Telemedicine Is not as Effective						95% Confidence Interval	
	*n*	*M* (*SD*)	*n*	*M* (*SD*)	*t*-test	df	*p*	Cohen’s *d*	LL	UL
Child Anxiety	7	52.87 (12.36)	2	74.35 (1.77)	2.34	7	0.052	1.87	−0.25	43.21
Child Depression	7	54.80 (8.46)	2	75.85 (1.91)	3.34	7	0.012	2.68	6.14	35.96
Parent Anxiety	7	57.63 (6.94)	2	75.60 (3.25)	3.43	7	0.011	2.75	5.58	30.37
Parent Depression	7	49.99 (8.83)	2	69.35 (2.62)	5.07	6.55	0.002	2.35	10.21	28.52

Note. Results from *t*-tests examining parent reports of child and parent mental health status (anxiety and depression) with perceived utility of telemedicine (telemedicine is as effective as face-to-face meetings vs. not effective).

## Data Availability

The data presented in this study are available on request from the corresponding author.

## Supplementary Materials

The following supporting information can be downloaded at: https://www.mdpi.com/article/10.3390/curroncol30020186/s1, Table S1: COVID-19 Study Questionnaire.
