# Supplementary material for: The Impact of the Early COVID-19 Global Pandemic on Children Undergoing Active Cancer Treatment and Their Parents
Source: Curr Oncol. 2023 Feb 17;30(2):2441–56. doi: 10.3390/curroncol30020186 (PMC9954946; doi:10.3390/curroncol30020186)
Supplement: Supplementary file 1 [file curroncol-30-00186-s001.zip › curroncol-2174244-SI.pdf]

Table S1. COVID-19 Study Questionnaire

1. Has any type(s) of your child's cancer treatments been affected as a result of COVID-19? Choose all that apply<br>- Chemotherapy<br>- Surgery<br>- Radiation<br>- Stem cell transplant<br>- Antibody treatment<br>- Immunotherapy<br>- Vaccines<br>- Supportive medications including pain medication<br>- Alternative medicines<br>- Mental health care<br>- Survivorship follow-up<br>- Palliative radiation<br>- Hospice care<br>- Outpatient clinic<br>- Intensive in-hospital therapy<br>- Scans (e.g., MRI, CT)<br>- Bloodwork	- Not applicable<br>- Not affected<br>- Delayed<br>- Modified<br>- Cancelled
2. Many institutions have moved to virtual telemedicine consultations to reduce the number of visits to the hospital clinics for both children in treatment as well as survivorship follow up. Did you or your child participate in a telemedicine appointment(s)?	- No<br>- Yes
3. Please rate your perception of the benefit of telemedicine.	- No benefit<br>- Little benefit<br>- Beneficial<br>- Very beneficial
4. Was the telemedicine consult most often with a member of the child's regular oncology team or was it with someone new?	- Member of the child's regular team<br>- Someone new
5. Did you feel the telemedicine meeting was as effective as a face-to-face meeting?	- Not at all<br>- Somewhat effective

	- Effective<br>- Very effective, better than face-to-face
6. Did you feel safe going to the hospital?	- Yes<br>- No<br>- N/A
7. Did your child feel safe going to the hospital?	- Yes<br>- No<br>- N/A
8. Is there a hospital policy limiting the number of people who can accompany your child to the hospital?	- Yes<br>- No<br>- N/A
9. If there is a hospital policy limiting the number of people who can accompany your child, what is the emotional impact on you/your child?	- No emotional impact<br>- Slight emotional impact<br>- Considerable negative emotional impact<br>- Significant negative emotional impact
10. Is there a hospital policy limiting who can accompany your child - for example no grandparent, siblings, friends?	- Yes<br>- No<br>- Don't know
11. If there is a hospital policy limiting the number of people who can accompany your child and who that individual may be, what is the emotional impact <u>on you</u>?	- No emotional impact<br>- Slight emotional impact<br>- Considerable negative emotional impact<br>- Significant negative emotional impact<br>- Not applicable
12. If there is a hospital policy limiting the number of people who can accompany your child and who that individual may be, what is the emotional impact <u>on your child</u>?	- No emotional impact<br>- Slight emotional impact<br>- Considerable negative emotional impact<br>- Significant negative emotional impact<br>- Not applicable
13. What COVID-19 restrictions are currently in place where your child lives? (Choose all that apply)	- School cancelled<br>- School being offered via remote learning (e.g., home schooling)<br>- Childcare cancelled or interrupted (e.g., daycare)<br>- Public gatherings limited to < 5 people<br>- Public gatherings limited to < 15 people<br>- Public gatherings limited to < 50 people<br>- Must wear mask/face covering in public<br>- Must maintain physical or social distance when in public

	- Curfew<br>- Stay at home order (except for essential work or outings)<br>- Community beginning to re-open stores and services (e.g., a multi-phase relaunch plan)<br>- No restrictions<br>- Other (please specify)
14. Have you been told by a doctor or other health care professional that <u>your child</u> has or had COVID-19 (novel coronavirus)?	- No<br>- Yes, and the condition is still present<br>- Yes, and the condition is no longer present
15. Has anyone in <u>your household</u> been diagnosed with COVID-19 (novel coronavirus)?	- No<br>- Yes, and the condition is still present<br>- Yes, and the condition is no longer present
16. Has <u>your child</u> been exposed to someone who has been diagnosed with COVID-19?	- No<br>- Yes
17. Compared to before the COVID-19 pandemic, how would you say <u>your child's</u> mental health is now?	- Much better now<br>- Somewhat better now<br>- About the same<br>- Somewhat worse now<br>- Much worse now
18. Do you think <u>your child's</u> current mental health is:	- About the same as family and friends<br>- Better than family and friends<br>- Worse than family and friends
19. Is your child's current mental health impacted by: (check all that apply)	- Tied to fears/worries about their cancer and treatment<br>- Tied to fears/worries about catching COVID-19<br>- Tied to the media and messaging about the COVID-19 pandemic<br>- Tied to fears/worries about the uncertainty related to the COVID-19 pandemic<br>- Tied to fears/worries about the uncertainty related to how COVID-19 might impact them as a survivor of childhood cancer<br>- Tied to feelings of isolation from peers<br>- Tied to difficulties with remote learning<br>- Other (Please specify)<br>- Not applicable, my child's mental health has not been impacted by the COVID-19 pandemic

20. Has your child ever had suicidal thoughts before the pandemic began?	- Yes<br>- No<br>- Unsure
21. How would you rate your current health?	- Poor<br>- Fair<br>- Good<br>- Very good<br>- Excellent
22. Compared to before the COVID-19 pandemic, how would you say your mental health is now?	- Much better now<br>- Somewhat better now<br>- About the same<br>- Somewhat worse now<br>- Much worse now
23. Is your current mental health impacted by: (check all that apply)	- Tied to fears/worries about your child's cancer and treatment<br>- Tied to fears/worries about catching COVID-19<br>- Tied to the media and messaging about the COVID-19 pandemic<br>- Tied to fears/worries about the uncertainty related to the COVID-19 pandemic<br>- Tied to fears/worries about the uncertainty related to how COVID-19 might impact them as a child with cancer<br>- Tied to feelings of isolation from peers<br>- Tied to difficulties with remote learning<br>- Tied to fears/worries about other factors in your life (i.e., boredom, feeling stuck at home)<br>- Other (Please specify)<br>- Not applicable, my mental health has not been impacted by the COVID-19 pandemic
24. Do you feel you are able to get the psychosocial support needed?	- Yes<br>- No
25. Do you feel your child is able to get the psychosocial support needed?	- Yes<br>- No
26. If you were previously engaged in mental health services, were services continued with the COVID-19 pandemic?	- Yes<br>- No<br>- I was not previously engaged in mental health services
